# Stem Cells for Cardiovascular Diseases Revisited in 2019

**DOI:** 10.21470/1678-9741-2019-0316

**Published:** 2019

**Authors:** Renato A. K. Kalil, Nance B. Nardi

**Affiliations:** 1 Instituto de Cardiologia do Rio Grande do Sul/Fundação Universitária de Cardiologia and Federal University of Health Sciences of Porto Alegre (UFCSPA), Porto Alegre, RS, Brazil. E-mail: kalil.renato@gmail.com

Therapeutic application of stem cells for untreatable cardiovascular diseases, such as refractory angina and myocardial failure, caused a frenzy in clinical research in the late 1990’s and early 2000’s. Experimental reports have shown marked improvements in myocardial contractility in heart failure models, and increased myocardial perfusion or even myocardial replacement after necrosis in ischemic models.

More than 200 clinical trials were produced, but the experimental effects could not be reproduced. Indeed, an improvement in cardiac function as well as some angiogenic reperfusion have been observed at the clinical level, but those effects were light and temporary, not sufficient to represent a usable therapeutic tool. The reasons for that are an actual challenge to researchers.

There are many hypotheses for stem cell therapy failure in clinical therapy. Animal experiments are done in young individuals and outcomes are evaluated invariably at short term. Cardiac diseases are present in older patients, in whom stem cells are also old and submitted to pharmacological effects of therapeutic drugs. Potent and prolonged improvements are necessary to influence clinical outcomes, differently from what can be achieved in animal research. Those could be some of several explanations.

The mechanism of action of stem cell therapy is also under exploration. The elements responsible for the effects need to be better understood. Cellular proliferation, paracrine effects, and delivery of cell elements or components are theories to be studied. Proliferation has been demonstrated as not feasible in clinical level.

Some considerations should be brought to mind.

There are two main types of stem cells. Pluripotent stem cells, capable of differentiating in any type of mature cells, of existing in the blastocyst, and can also be produced by genetic reprogramming (induced pluripotent stem cells, or iPS cells)^[1]^. And adult or somatic stem cells, which exist in all organs and are responsible for maintenance and repair of adult tissues. The therapeutic potential of both types of stem cells in heart diseases is under investigation.

The therapeutic use of adult stem cells started over 50 years ago, with bone marrow blood transplantation for hematological diseases. During the last 20 years, the potential of adult stem cells to treat non-hematological diseases has been widely investigated, and the mesenchymal stem cell (MSC) has emerged as a promising therapeutic candidate in regenerative medicine.

MSCs are undifferentiated cells able to self-renew and to give rise to cells with mature mesenchymal phenotypes. Although they are more usually isolated from the bone marrow and, more recently, from adipose tissue, they reside in virtually all tissues, where they have an active role in the repair of focal injuries^[2]^. When MSCs are isolated from the organism and cultivated in vitro, which is usually necessary for therapeutic use, they lose their stemness, which led to a proposal to change their name to multipotent mesenchymal stromal cells. Their therapeutic effect is exerted mainly by the secretion of bioactive molecules with supportive, antiapoptotic, angiogenic, chemoattractant, antiscarring, and immunomodulatory functions. Extracellular vesicles, sized 80-1000 nm (microvesicles) and 50-200 nm (exosomes), were described as the mediating factor in MSC secretion^[3]^.

MSCs are the most widely used cells in regenerative medicine. A search in the clinicaltrials.gov database shows more than 1,000 clinical trials using this cell type. A recent review^[4]^ described the use of MSCs in orthopedic, degenerative, autoimmune, and inflammatory diseases, as well as in immune rejection of allogeneic transplantation. Results show that this type of cell therapy is safe, but actual data on its efficacy are often preliminary.

In the case of heart diseases, the first animal studies showed improved cardiac function after cell therapy, and a great number of preclinical trials were performed with similar results. Bone marrow cells were used in most cases, but soon the existence of endogenous cardiac stem cells (CSCs), known as c-kit+ cells, was proposed by Piero Anversa's group. Later work by other groups have casted doubt on the existence of a CSC, and more recently Anversa et al. had papers retracted or with “expression of concern” by the publishers^[5]^. The retraction of those papers as result of a Harvard University investigation of scientific misconduct produced a strong negative impact in the field. But, also, could have explained why most of the results by that group could not be reproduced by others.

A great number of clinical trials for heart diseases, using bone marrow or cardiac cells, have been completed, are ongoing, or have been approved worldwide, including reports from our group^[6]^. The results uniformly show that there are mild and transitory effects in myocardial improved contraction and perfusion, and they are not sufficient to change clinical outcomes.

There are still paths to be explored, however. Pluripotent stem cells can be differentiated in vitro in any type of functional cell, and then used for tissue repair. Menasché, one of the first groups to publish clinical series of stem cell therapy in cardiac diseases, reported a recent 18-month phase I study demonstrating safety and increased systolic function in heart failure patients after transplantation of embryonic stem cell-derived cardiovascular progenitor cells embedded in fibrin patch^[7]^. iPS cells, however, are the pluripotent stem cells more extensively studied, due to the easiness of production and mainly to the fact that they are genetically identical to the donor patient. Their use in disease modelling and cell replacement therapy is under intensive study, but still emerging in the clinical setting^[8]^. A summary of our experience on stem cell research has been recently published^[9]^.

Another path is the exploration of what has been named as “cell-free stem-cell therapy”, a series of reports that study the mechanisms of action of cell-derived elements, like extracellular microvesicles, exosomes, mitochondria, proteins, nucleic acids, and possibly others, which could be isolated, purified, concentrated, and applied over the failing or ischemic myocardium. Besides being more efficient, those elements might have significant practical advantages over intact cells, as they can be managed similarly to pharmacological drugs, produced and stored in quantity for timely administration^[10]^.

In summary, despite the great attention, research time and resources invested in clinical trials using MSCs or CSCs for heart diseases, which are still going on, a consensus is building on the inadequacy of this therapeutic approach. The scientific community is being urged to "refocus the attention of the cardiac regeneration field on more promising approaches"^[11]^. Stem cells may still be a great solution, but not by the transplantation of adult cells. The approaches of iPS cells and cell-free therapy are under study and could bring answers in the future.
